# Supplementary material for: PrimSeq: A deep learning-based pipeline to quantitate rehabilitation training
Source: PLOS Digit Health. 2022 Jun 16;1(6):e0000044. doi: 10.1371/journal.pdig.0000044 (PMC9681023; doi:10.1371/journal.pdig.0000044)
Supplement: S1 Table — These representative rehabilitation activities were used to generate an abundant sample of functional primitives for model training. Activity parameters include the workspace setup, target objects, and instructions to complete each task. The table and counter edges are their anterior edges closest to the patient. Patients could perform the actions within the activity in their preferred order. (DOCX) [file pdig.0000044.s001.docx]

| Activity | Workspace | Target Object(s) | Instructions to Patient |
| --- | --- | --- | --- |
| Shelf task | On table, two Plexiglas shelves (length: 90 cm; width: 25.5 cm; height: 33, 53 cm) with three targets (22.5, 45, and 67.5 cm from left-most edge) placed 20 cm from table edge and center target (diameter: 5 cm) placed 2.5 cm from table edge | Toilet paper roll wrapped in self-adhesive wrap | Move the toilet paper back and forth between the center target and each target on the shelf, returning the arm to rest between each motion and the end. |
| Tabletop task | On table, horizontal circular array (diameter: 48.5 cm) of eight outer targets and center target (diameter: 5 cm) placed 2.5 cm from table edge | Toilet paper roll wrapped in self-adhesive wrap | Move the roll back and forth between the center and each outer target, returning the arm to rest between each motion and the end. |
| Feeding | On table, paper plate (diameter: 21.6 cm) placed at midline, 5 cm from table edge; utensils placed three cm from table edge and five cm from either side of plate; plastic sandwich baggie containing a slice of bread placed 25 cm from table edge, and 23 cm left of midline; and margarine packet placed 32 cm from table edge and 17 cm right of midline | Paper plate, fork, knife, re-sealable sandwich baggie, slice of bread, single-serve margarine packet | Pick up the sandwich baggie and open it, remove the bread and put it on the plate, open the margarine packet and spread margarine on the bread, cut the bread into four pieces, cut off and eat a small piece. |
| Drinking | On table, water bottle and paper cup placed 18 cm left and right of midline, respectively, and 25 cm from table edge | Water bottle (12 oz), cup (four oz) | Pick up the water battle and open it, pour some water into the cup, take a sip of water, place the cup on the table, and replace the cap on the bottle. |
| Combing hair | On table, comb placed at midline, 25 cm from table edge | Comb | Pick up the comb and comb both sides of the head. |
| Donning glasses | On table, pair of glasses placed at midline, 25 cm from table edge | Pair of glasses | Pick up the glasses, open the sides, put on the glasses, briefly place your hands down on the table, and then remove the glasses and place them on the table. |
| Applying deodorant | On table, deodorant placed at midline, 25 cm from table edge | Deodorant (solid, twist-base) | Pick up the deodorant, remove the cap, twist the base a few times, apply the deodorant to each armpit, replace the cap, untwist the base, place the deodorant on the table. |
| Washing face | At counter, small tub (length: 32.3 cm; width: 24.1 cm; depth: 2.5 cm) in sink; two folded washcloths on each side of counter next to sink, 30 cm from counter edge | Washcloths, tub, faucet handles | Fill the tub with water, take one washcloth and dip it into water, wring it, wipe each side of the face, and place it back on the counter. Then use the other washcloth to dry the face, and place it back on the counter. |
| Brushing teeth | At counter, toothpaste and toothbrush placed on either side of the sink, 30 cm from counter edge | Travel-sized toothpaste, toothbrush with built-up foam grip, faucet handles | Wet the toothbrush, open the toothpaste and apply it to the toothbrush, replace the cap on the toothpaste tube, brush teeth, rinse the toothbrush and mouth, and place the toothbrush back on the counter. |
