# Supplementary material for: PrimSeq: A deep learning-based pipeline to quantitate rehabilitation training
Source: PLOS Digit Health. 2022 Jun 16;1(6):e0000044. doi: 10.1371/journal.pdig.0000044 (PMC9681023; doi:10.1371/journal.pdig.0000044)
Supplement: S2 Table — The motion capture system (myomotion, Noraxon, USA) used 9 IMUs and a proprietary height-scaled model to generate 22 upper body angles, shown in relation to their joint of origin. ‡Shoulder total flexion is a combination of shoulder flexion/extension and shoulder ad-/abduction. *Thoracic angles are computed between the cervical (C7) and thoracic (T10) vertebrae. †Lumbar angles are computed between the thoracic vertebra and pelvis. (DOCX) [file pdig.0000044.s002.docx]

| Joint | Anatomical angle |
| --- | --- |
| Shoulder | Shoulder flexion/extension  Shoulder internal/external rotation  Shoulder adduction/abduction  Shoulder total flexion^‡^ |
| Elbow | Elbow flexion/extension |
| Wrist | Wrist flexion/extension  Forearm pronation/supination  Wrist radial/ulnar deviation |
| Thorax | Thoracic* flexion/extension  Thoracic* axial rotation  Thoracic* lateral flexion/extension |
| Lumbar | Lumbar^‡^ flexion/extension  Lumbar^‡^ axial rotation  Lumbar^‡^ lateral flexion/extension |
